# Supplementary material for: Ethanolic Extract from Fruits of Pintoa chilensis, a Chilean Extremophile Plant. Assessment of Antioxidant Activity and In Vitro Cytotoxicity
Source: Plants (Basel). 2024 May 18;13(10):1409. doi: 10.3390/plants13101409 (PMC11125100; doi:10.3390/plants13101409)
Supplement: Supplementary file 1 [file plants-13-01409-s001.zip › plants-2975752-supplementary.pdf]

Table S1. Collection coordinates of the *P. chilensis* plant in the province of Copiapó, Atacama Region, Chile.

Family	Genus	Collection location	Collection date
Zygophyllaceae	*Pintoa chilensis* Gay 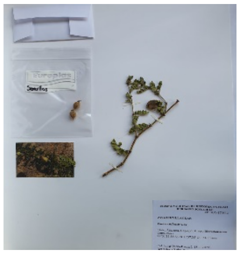	Biorestauración consultores<br>Provincia de Copiapó<br>Región de Atacama<br><br>548 m n.s.m	15-11-2020

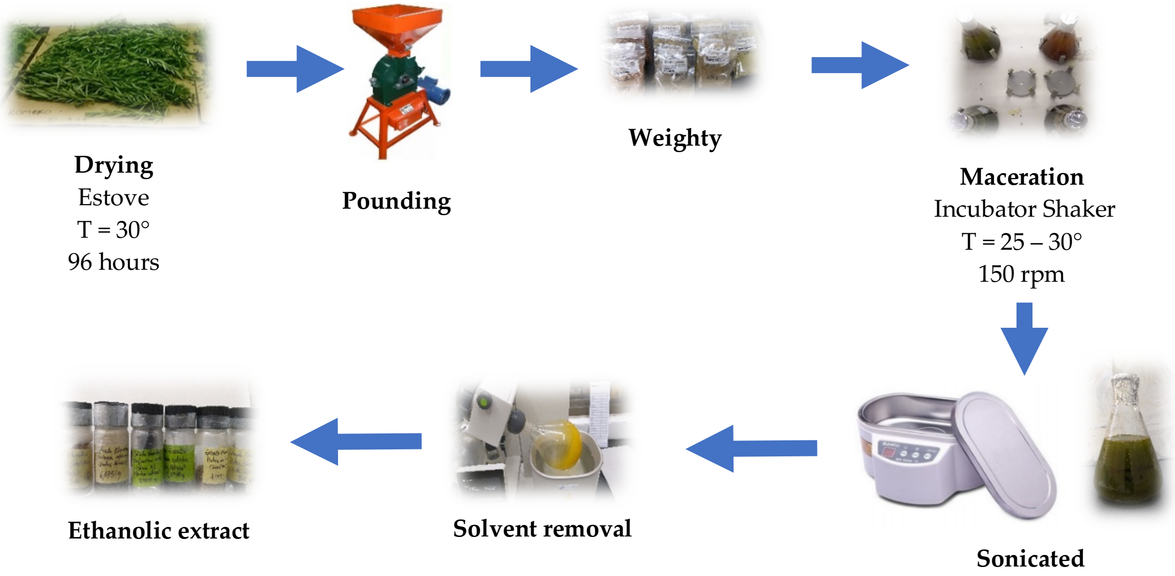

Figure S1. Preparation of ethanolic extract from *Pintoa chilensis (ap* and *f)*.

Table S2. Percentage yield of the ethanol extract obtained from the *P. chilensis* plant.

Plant	Initial Mass	Final Mass	Yield
P. chilensis (ap)	50.07 g	21.92 g	43.80%
P. chilensis (f)	50.08 g	15.25 g	30.45%

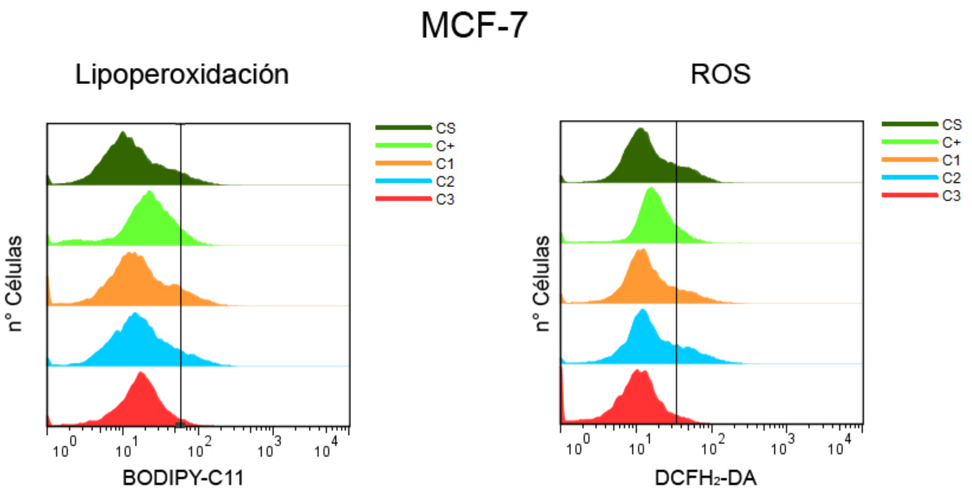

Figure S2. a) Histogram showing lipoperoxidation levels measured for the MCF-7 tumor cell line by flow cytometry with BOCIPY-C11 induced by the extract EF2. b) Histogram showing ROS levels measured for the MCF-7 tumor cell line by flow cytometry with $DCFH_2$-DA induced by the extract EF2.

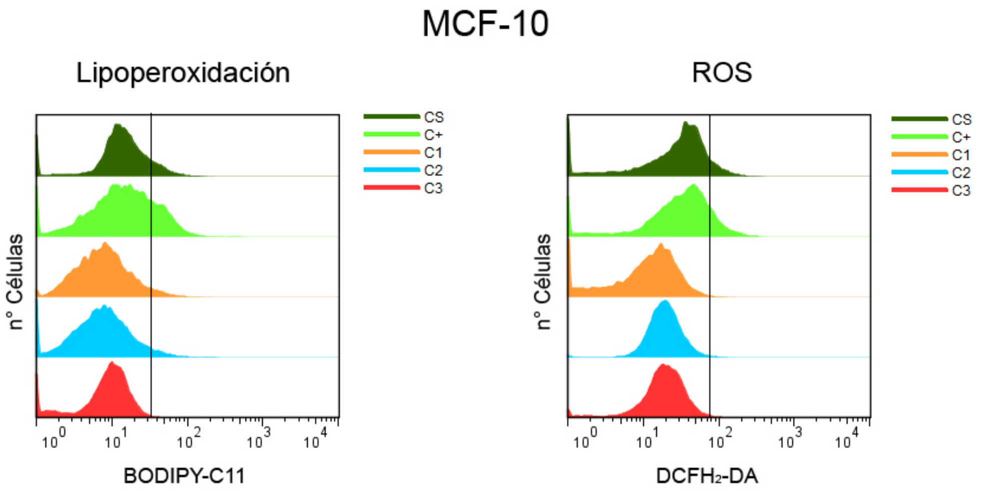

Figure S3. a) Histogram showing lipoperoxidation levels measured for the MCF-10A non-tumoruos cell line by flow cytometry with BOCIPY-C11. b) Histogram showing ROS levels measured for the MCF-10A non-tumoruos cell line by flow cytometry with $DCFH_2$-DA.

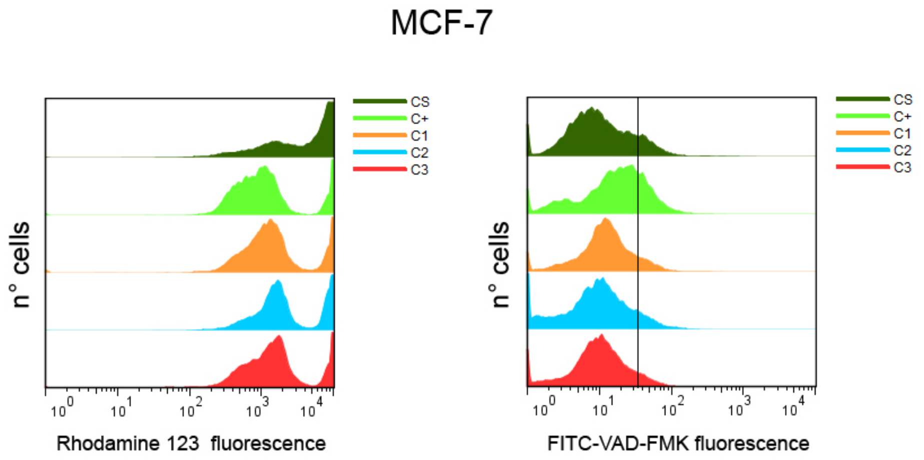

Figure S4. a) Histogram showing effect of mitochondrial membrane permeability in MCF-7 tumor cell line by flow cytometry with Rhodamine-123 (Rh-123) induced by the extract EF2. b) Histogram showing effect of caspase activation in MCF-7 tumor cell line by flow cytometry with FITC-VAD-FMK induced by the extract EF2.

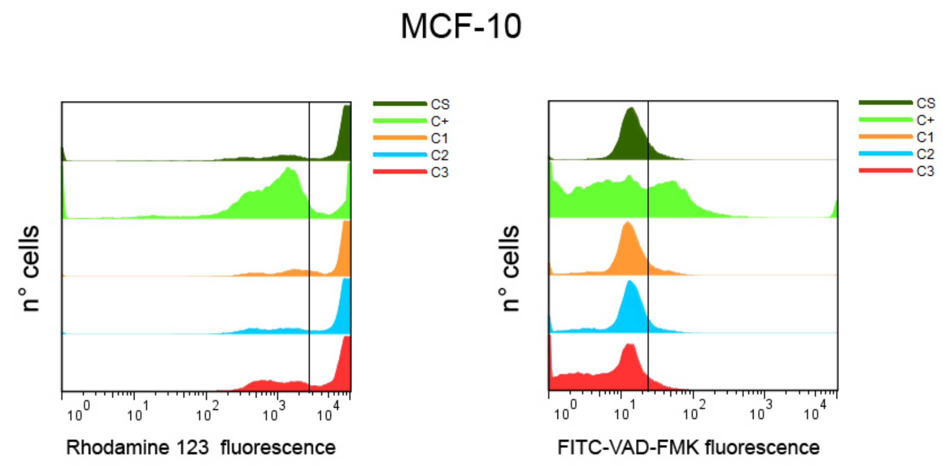

Figure S5. a) Histogram showing effect of mitochondrial membrane permeability in MCF-10A non-tumoruos cell line by flow cytometry with Rhodamine-123 (Rh-123). b) Histogram showing effect of caspase activation in in MCF-10A non-tumoruos cell line by flow cytometry with FITC-VAD-FMK.

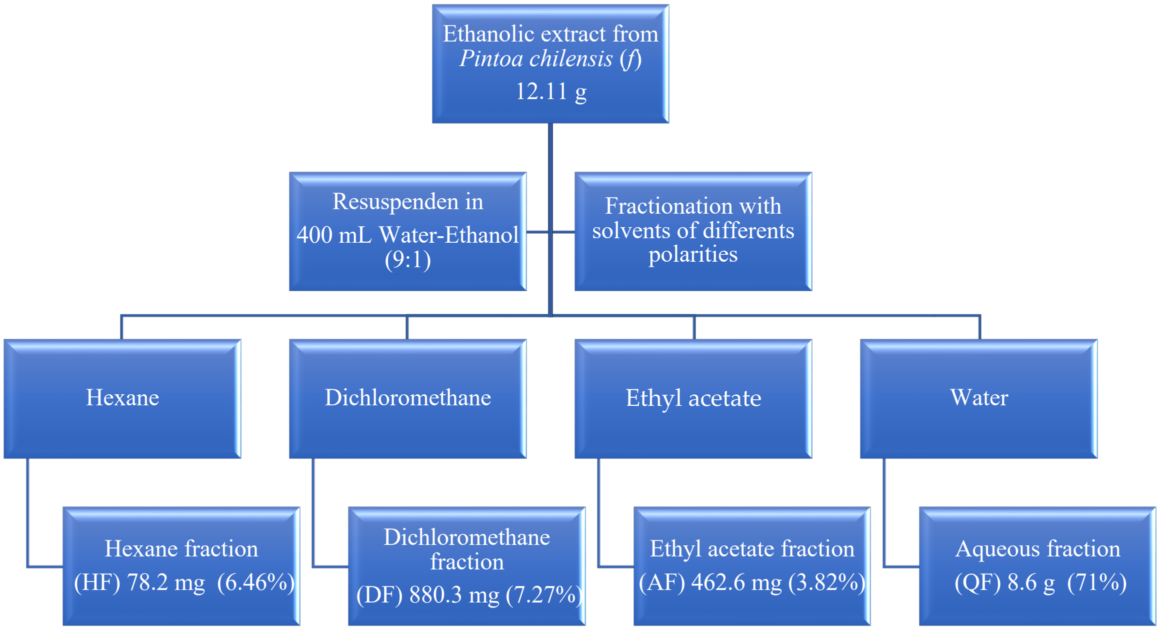

Figure S6. Liquid-liquid fractionation of ethanolic extract from *Pintoa chilensis (f)*.

Table S3. GC-MS analysis for the dichloromethane fraction (DF) obtained from the ethanolic extract of *P. chilensis (f).*

No.	RT	Main compounds	RI	RI ref	match	% Area
1	7.6474	Unknown	974	-	-	1.35
2	9.2152	Unknown	1043	-	-	0.43
3	9.4669	1-3-propanediol-di-TMS	1053	1073	928	0.48
4	9.5985	Unknown	1058	-	-	0.79
5	12.425	Unknown	1136	-	-	0.08
6	14.125	Urea, N,N'-bis(TMS)- (CAS)	1169	1243	899	0.19
7	15.235	3,7-Dioxa-2,8-disilanonane, 2,2,8,8-tetramethyl-5-[(TMS)oxy]	1191	1292	882	3.65
8	15.858	Unknown	1255	-	-	0.45
9	20.356	Butanedioic acid, [(TMS)oxy]	1497	-	860	0.08
10	21.386	Trimethyl(2,6 diter-butylphenoxy) silane	1544	-	792	0.26
11	24.51	2-Methylresorcinol, bis(trimethylsilyl) ether	1690	1455	707	0.49
12	25.105	Levoglucosan, tris(trimethylsilyl)	1619	1697	856	0.09
13	25.649	Xylitol, 1,2,3,4,5-pentakis-O-(trimethylsilyl)-	1646	1744	748	0.13
14	26.473	Tagatofuranose, pentakis(trimetthylsilyl) ether	1787	1813	767	0.32
15	27.171	Unknown	1723	-	-	0.20
16	27.605	Myristic acid-monotms	1745	1842	888	3.52
17	27.92	D-Pinitol, pentakis(trimethylsilyl) ether	1762	1815	894	7.11
18	28.121	Unknown	1773	1744	615	0.68
19	28.647	Xylitol, 1,2,3,4,5-pentakis-O-(trimethylsilyl)	1900	2153	685	0.29
20	28.796	Hexopyranose, 1,2,3,4,6-pentakis-O-(pentakis)	1808	-	665	0.20
21	29.002	Myoinositol TMS	1819	1930	913	1.17
22	29.231	D-Altrose, 2,3,4,5,6-pentakis-O-(Trimetyhlsilyl)	1831		829	0.13
23	29.448	4,6-Pteridinediol, 2-trimethylsilamino-, O,O-bis(trimethylsilyl)-, ether	1843	2179	674	0.45
24	29.826	Ethyl 2,3,4,6-tetrakis-O-(TMS)-D-Glucopyranoside	1864	2027*	848	0.22
25	30.701	β-D-Allopyranose, pentakis(trimethylsilyl) ether	1912	1829	902	1.32
26	31.176	Hexadecanoic acid, trimethylsilyl ester	1939	2039	940	0.88
27	31.279	Unknown	1945			0.16
28	32.155	D-(+)-Turanose, octakis(trimethylsilyl) ether	2094	2693	722	0.18
29	32.618	D-(+)-Galacturonic acid, O-pentakis(trimethylsilyl) deriv.	2022	1943	674	0.22
30	33.35	4-Nitrophenyl-β-D-galacturonide, tris(trimethylsilyl) ether	2065	2837	676	0.35
31	33.837	d-Glucopyranose, 1-C-octyl-2,3,4,6-tetra-O-trimethylsilyl	2194	2698*	673	0.23
32	34.071	Lauric acid, 2,3-bis(trimethylsiloxy)propyl ester	2108	2227	788	0.14
33	34.512	Octadecanoic acid, trimethylsilyl ester	2235	2236	926	0.87
34	37.104	Myristic acid, 2,3-bis(trimethylsiloxy)propyl ester	2399	2424	899	0.31
35	39.496	2-Monopalmitoylglycerol trimethylsilyl ether	2557	2576	880	1.36
36	40.034	Hexadecanoic acid, 2,3-bis[(trimethylsilyl)oxy]propyl ester	2596	2613	855	5.74
37	41.43	Sucrose, octakis(trimethylsilyl) ether	2696	2610	950	0.89

Table S3. Continuation….

No.	RT	Main compounds	RI	RI ref	match	% Area
38	42.168	2-Monostearin trimethylsilyl ether	2751	2775	895	1.31
39	42.265	Unknown	2758	-	-	0.19
40	42.683	Octadecanoic acid, 2,3-bis[(trimethylsilyl)oxy]propyl ester	2789	2808	856	4.68
41	44.136	Unknown	2900	-	-	0.38
42	45.201	Unknown	2985	-	-	0.14
43	50.78	Unknown	3532	-	-	2.89

[a] RI: Retention indexes relative to C_7-C_{40} n-alkanes on the BP-5MS capillary column. [b] Retention index reported in the literature.

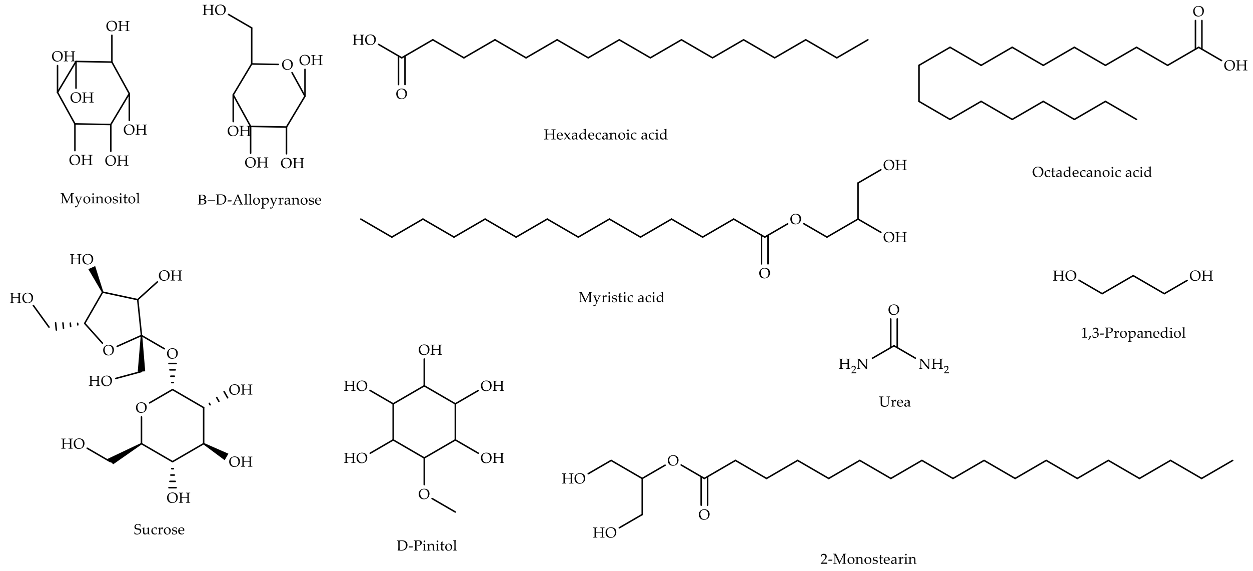

Figure S7. Chemical structures of compounds detected in GC-MS analysis of dichloromethane fraction obtained from ethanolic extract from *Pintoa chilensis (f)*.
